# Characterization of the Ubiquitylating Components of the Human Malaria Parasite’s Protein Degradation Pathway

**DOI:** 10.1371/journal.pone.0043477

**Published:** 2012-08-17

**Authors:** Duk-Won D. Chung, Nadia Ponts, Jacques Prudhomme, Elisandra M. Rodrigues, Karine G. Le Roch

**Affiliations:** 1 Department of Cell Biology and Neuroscience, University of California Riverside, Riverside, California, United States of America; 2 INRA, MycSA UR 1264, BP81, 33883 Villenave d’Ornon Cedex, France; Jr. Johns Hopkins Bloomberg School of Public Health, United States of America

## Abstract

Ubiquitin-dependent protein degradation within malarial parasites is a burgeoning field of interest due to several encouraging reports of proteasome inhibitors that were able to confer antimalarial activity. Despite the growing interest in the *Plasmodium* proteasome system, relatively little investigation has been done to actually characterize the parasite degradation machinery. In this report, we provide an initial biological investigation of the ubiquitylating components of the endoplasmic reticulum-associated degradation (ERAD) system, which is a major pathway in targeting misfolded proteins from the ER to the cytosol for proteasome degradation. We are able to show that the ERAD system is essential for parasite survival and that the putative *Plasmodium* HRD1 (E3 ubiquitin ligase), UBC (E2 ubiquitin conjugating enzyme) and UBA1 (E1 ubiquitin activating enzyme) are able to mediate *in vitro* ubiquitylation. Furthermore, by using immunofluorescence, we report that *Plasmodium* HRD1 localizes to the ER membranes, while the *Plasmodium* UBC and UBA1 localize to the cytosol. In addition, our gene disruption experiments indicate that the *Plasmodium* HRD1 is likely essential. We have conducted an initial characterization of the ubiquitylating components of the *Plasmodium* ERAD system, a major pathway for protein degradation and parasite maintenance. In conjunction with promising proteasome inhibitor studies, we explore the possibility of targeting the *Plasmodium* ERAD system for future bottom-up drug development approaches.

## Introduction

Malaria is one of the deadliest infectious diseases of the world, infecting up to half a billion and killing up to one million people each year [1]. Though progresses were made in combating malaria with multi-drug therapies [2], the high-cost treatment and the rise of drug resistances beckon the need for novel and cheap antimalarials. The causative agents of malaria are protozoan parasites that belong to the genus *Plasmodium*. Among them, *Plasmodium falciparum* is the deadliest human-infecting species. With the urgent need of developing new anti-malarial therapies, there has been much effort in understanding how the *Plasmodium* regulates its life cycle to subsequently identify potential new targets for drug development and therapeutic intervention.

In recent years, several studies showed that proteasome inhibitors have significant antimalarial properties [3]–[7] (see [8] for a review). Previous studies characterized some of *P. falciparum*’s 26S proteasome components [9], [10] and a bacteria-like proteasome [11]–[13], both involved in protein degradation. Though the protein degradation *via* the 26S proteasome closely involves ubiquitylation of target substrates, very little work has been done regarding the characterization of the parasite’s ubiquitylating machinery acting upstream of the proteasome, possibly leading to the discovery of parasite-specific divergences that can be exploited for drug targeting. In eukaryotes, the endoplasmic reticulum-associated degradation (ERAD) pathway mediates protein degradation during quality control. Aberrant proteins are recognized by ER luminal chaperone proteins and protein disulfide isomerases to help discriminate properly folded proteins from misfolded proteins [14]. Misfolded proteins are shuttled to the DER1 translocon complex, which forms a hydrophobic pore to allow the retro-translocation of proteins through the ER membrane. Within this translocon complex, the HRD1 E3 ubiquitin ligase interacts with membrane-bound proteins needed for retro-translocation and helps form the hydrophobic pore complex [15]. The HRD1 E3 enzyme also catalyzes, with the intervention of other ubiquitylating enzymes, the ubiquitylation of the target misfolded protein that is the prerequisite for subsequent retro-translocation to the cytosol and destruction by the 26S proteasome [16]–[18]. Typically, ubiquitylation involves the covalent attachment of a ubiquitin moiety to lysine residues of protein substrates *via* the hierarchical intervention of an E1 ubiquitin-activating enzyme, an E2 ubiquitin-conjugating enzyme, and an E3 ubiquitin ligase that is usually involved in specific substrate recognition [19], [20]. Up until now, no functional study has investigated the ubiquitylating components that compose the malaria parasite ERAD pathway. While ubiquitin E1 and E2 enzymes seem to be well conserved across eukaryotic phyla, *Plasmodium* E3 ubiquitin ligases have been shown to have high levels of divergences that can be utilized for the development of new antimalarials [21].

Here, we present the identification and biochemical characterization of the ubiquitylating components of the *P. falciparum* ERAD system: one ubiquitin-activating E1 enzyme, an ubiquitin-conjugating E2 enzyme, and an ubiquitin E3 ligase. Our results show that the *Plasmodium* ERAD system is vital to the parasite and that recombinant E1, E2, and E3 enzymes promote ubiquitylation *in vitro*. Furthermore, immunofluorescence microscopy experiments also reveal that E1 and E2 localize to the cytosol whereas the E3 ubiquitin ligase is found within the ER membrane, consistent with their respective functions in the ERAD pathway. Finally, gene disruption experiments suggest that the ubiquitylating components of the *Plasmodium* ERAD system are essential to the parasite survival. This analysis contributes to our understanding of the mechanisms leading to protein degradation in the human malaria parasite and proposes the components of the ERAD pathway as potential targets for the ‘bottom-up’ development of new drugs.

## Results

### ERAD-specific Inhibitor Eeyarestatin I Validates the Essentiality of an Ubiquitin-dependent ERAD System in *Plasmodium*

**Figure 1:**
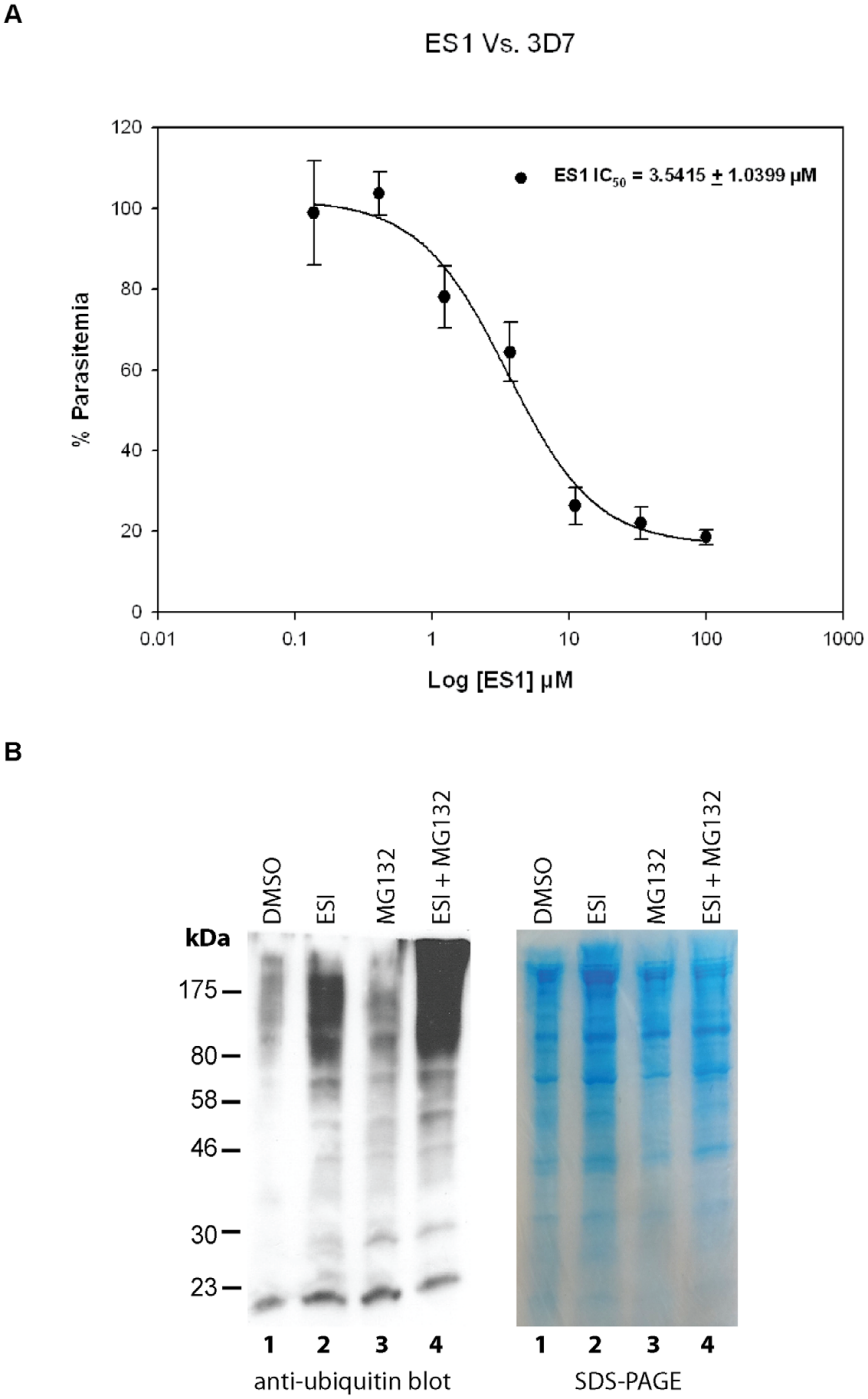
ERAD inhibitor Eeyarestatin I inhibits *P. falciparum in vitro* and increases the amount of ubiquitylated *Plasmodium* proteins. (**A**) Eeyarestatin I (ES_1_) inhibits *P. falciparum* 3D7 strain with an IC_50_ value of 3.5415±1.0399 µM (mean value ± standard error). Dose-response curve is plotted with increasing concentrations of ES_1_ (**B**) ES_1_ treatment leads to an accumulation of ubiquitylated proteins in *P. falciparum*. Anti-ubiquitin immunoblots show higher levels of ubiquitylated protein levels in *P. falciparum* parasites when treated with ES_1_ (lane 2) compared to the control (DMSO, lane 1). As a comparison, parasite were also treated with proteasome inhibitor MG132 [48] (lane 3) and exhibited slightly higher amounts of ubiquitylated proteins. When parasites were treated with both ES_1_ and MG132 (lane 4), significant increases in ubiquitylated products were detected.

To test the essentiality of the ERAD pathway in *Plasmodium*, we decided to test the inhibitory effects on *P. falciparum* cultures with Eeyarestatin I (ES_I_). ES_I_ a known ERAD inhibitor which acts to block the release of ubiquitylated misfolded proteins from the Sec61 translocon at the ER membrane [22], [23] thus preventing misfolded proteins from reaching the proteasome. *P. falciparum* parasites were found to be highly sensitive to ES_I_, with an IC_50_ of 3.5415±1.0399 µM (Figure 1A). In addition, we validated ES_I_’s ability to block the *Plasmodium* ERAD system by analyzing changes in the levels of ubiquitylated substrates between untreated and treated cultures. Parasites that were treated with ES_I_ were found to have heightened abundances of ubiquitylated proteins (Figure 1B). The observed accumulation of ubiquitylated proteins is likely the result of ES_I_ preventing the translocation of ubiquitylated misfolded proteins across the ER membrane for degradation as seen in other organisms [23]. These ES_I_ studies give preliminary indication that *Plasmodium* does indeed contain an ubiquitin-dependent ERAD system that is integral to parasite survival.

### *In silico* Analysis of the *Plasmodium* ERAD Ubiquitylating Components

The *P. falciparum* genome was scanned to identify candidates for the ubiquitylating components of the ERAD system based on sequence homology and domain architecture using a previously published approach [21]. We identified PFL1245w as the putative PfUba1 E1 ubiquitin enzyme, PFL0190w, as the putative PfUbc7 E2 ubiquitin conjugating enzyme, and PF14_0215 as a putative E3 ubiquitin ligase with distant protein homology with the human and yeast HRD1.

**Figure 2:**
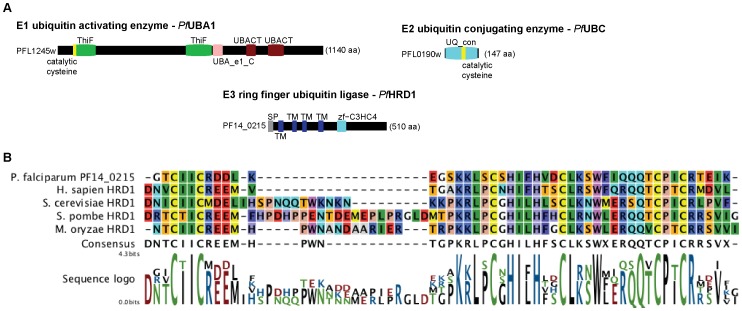
Domain architecture of the ubiquitylating components of the *Plasmodium* ERAD system and cf-C3HC4 domain alignment of PF14_0215. (**A**) Proteins are represented as black boxes of length proportional to their sizes in amino acids (aa). SP =  signal peptide; TM = transmembrane domain; ThiF/UBACT = repeats found in E1 ubiquitin-activating enzymes; UBA_e1_C = E1 ubiquitin-activating enzyme catalytic domain; UQ_con = E2 ubiquitin-conjugating enzyme domain; zf-C3HC4 = E3 ubiquitin ligase RING finger domain. Catalytic cysteines are marked in yellow. (**B**) Using MUSCLE [43], we performed protein alignments on PF14_0215 and found that its zf-C3HC4 domain had high homology to other previously characterized HRD1 of various model systems.

Similar to yeast and human UBA1, the 1140 amino acid (aa) long *Plasmodium* protein encoded by PFL1245w contains a ubiquitin-activating enzyme active site, two ubiquitin-like activating enzyme catalytic domains, two ThiF repeats, and a catalytic cysteine at the N-terminal end (Figure 2A). The short protein (147 aa in length) encoded by PFL0190w has strong homology to the human and yeast UBC7 and contains an ubiquitin conjugating enzyme domain taking up almost its whole length (Figure 2A). Finally, the protein encoded by PF14_0215 presents some sequence similarities with the canonical HRD1 of the ERAD pathway in eukaryotic models [21], [24], [25]. Similar to HRD1, PF14_0215 has multiple transmembrane domains, an E3 RING zinc finger (zf-C3HC4) domain on its C-terminal half, and a predicted signal peptide consistent with ER targeting (Figure 2A). The presence of four transmembrane domains is compatible with an ability to form pores to participate in the recognition and translocation of misfolded proteins across the ER membrane. Based on protein alignments, we show that the PF14_0215 protein contains a highly conserved zf-C3HC4 domain (289–336 aa) similar to that of other characterized HRD1 proteins from various eukaryotic model systems (Figure 2B).

Based on our *in silico* predictions, it is likely that these proteins have the necessary components to promote ubiquitylation and have enough homology to the human and yeast ERAD components to be part of the parasite’s ERAD system. In the following sections, we conduct some initial biological characterizations to validate our *in silico* data analyses.

### PF14_0215 is an Essential ER Membrane-bound E3 Ubiquitin Ligase

**Figure 3:**
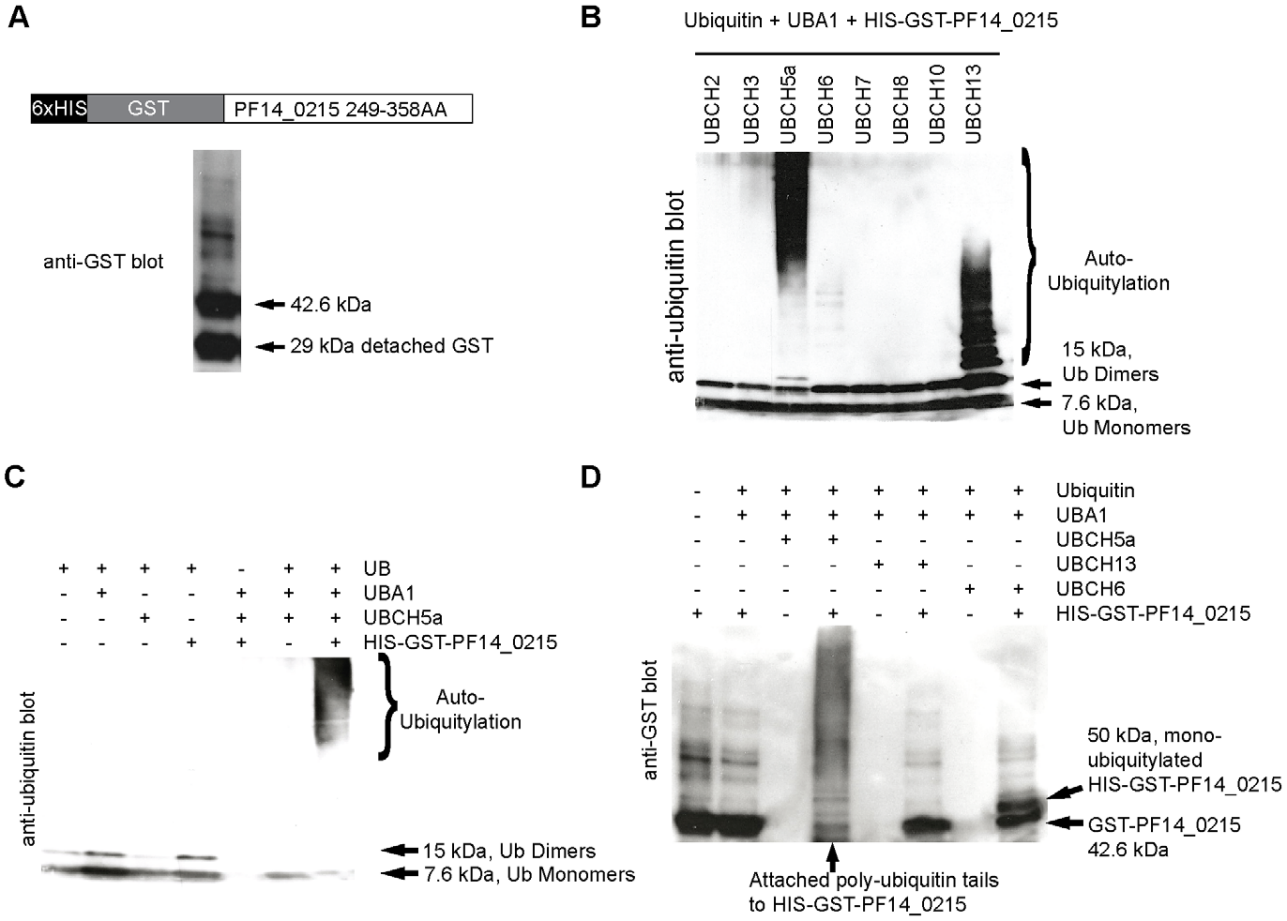
*In vitro* ubiquitylation assay of *Pf*HRD1 (PF14_0215). (**A**) Epitope-tagged recombinant *Pf*HRD1 was expressed in *E. coli* and purified. Anti-GST immunoblot on purified protein extracts reveals the presence of detached free GST tags (MW = 29 kDa) and of *Pf*HRD1-GST (MW = 42.6 kDa). (**B**) *In vitro* ubiquitylation assays were performed with commercial ubiquitin (Ub), human E1 UBA1, varying human E2 UBC enzymes (*i.e.,* UBCH2, UBCH3, UBCH5a, UBCH6, UBCH7, UBCH8, UBCH10, and UBCH13), and our purified extract of epitope-tagged recombinant *Pf*HRD1. Ubiquitylation patterns were revealed on anti-ubiquitin immunoblots. Different patterns of auto-ubiquitylation are observed with UBCH5a, UBCH13, and UBCH6. (**C**) *In vitro* ubiquitylation assays were performed in the presence (+) or absence (-) of commercial ubiquitin (UB), human E1 UBA1, human E2 UBCH5a, and our purified extract of epitope-tagged recombinant *Pf*HRD. Ubiquitylation patterns were revealed on anti-ubiquitin immunoblots. Ubiquitylation is specifically observed only when all components are mixed together, including *Pf*HRD1. (**D**) *In vitro* ubiquitylation assays were performed with commercial ubiquitin (Ub), human E1 UBA1, the human E2 enzymes UBCH5a, UBCH6, and UBCH13, and our purified extract of epitope-tagged recombinant *Pf*HRD1. The attachment of ubiquitin on the recombinant *Pf*HRD1 itself was detected by anti-GST immuno-detection. *Pf*HRD1 is poly-ubiquitylated in the presence of UBCH5a and mono-ubiquitylated in the presence of UBCH6 whereas UBCH13 does not permit *Pf*HRD1 auto-ubiquitylation.

Initiating our biological validation, we investigated the biochemical activity and the cellular localization of the protein encoded PF14_0215. First, the predicted RING domain of PF14_0215 was cloned into *E. coli*, fused to tandem GST and 6xHis tags, expressed, and purified (Figure 3A and Figure S1). *In vitro* ubiquitylation assays were performed by incubating the purified recombinant PF14_0215 RING domain with commercially available human E1 (UBA1), various human E2 (UBC) enzymes, and commercial purified ubiquitin (Figure 3B). Anti-ubiquitin immunoblots show that different patterns of ubiquitylation were obtained when our recombinant E3 and the commercial E1 were mixed with UBCH5a, UBCH6, or UBCH13_complex_. With UBCH5a, recombinant PF14_0215 RING domain elicited a laddering effect that mostly began from the 50 kDa range, while with UBCH13, the ubiquitin laddering began around the 15 kDa mark. Ubiquitylation was no longer detected when our recombinant PF14_0215 was removed from the assay (Figure 3C), which demonstrates that PF14_0215 promotes ubiquitylation. We investigated further the differences in ubiquitylating patterns obtained when different E2 enzymes are used. Anti-GST immunoblots of our *in vitro* ubiquitylation products were performed to distinguish auto-ubiquitylation events, a characteristic of RING E3 ligases, from the production of free poly-ubiquitin tails (Figure 3D). The high molecular weight banding pattern that was obtained in the presence of UBCH5a indicates the auto poly-ubiquitylation of our epitope-tagged PF14_0215. On the contrary, the single 8 kDa molecular weight shift of recombinant PF14_0215 obtained in the presence of UBCH6 indicates that only mono-ubiquitylation occurs. Finally, no auto-ubiquitylation of PF14_0215 was detected in the presence of UBCH13_complex_ (pattern not different from what obtained with our E3 alone). The previously observed pattern of ubiquitylation consisted of free poly-ubiquitin tails only (Figure 3B). Taken together, our results demonstrate that PF14_0215 is a genuine E3 ubiquitin ligase that catalyzes both the formation of poly-ubiquitin tails and its auto-ubiquitylation.

**Figure 4:**
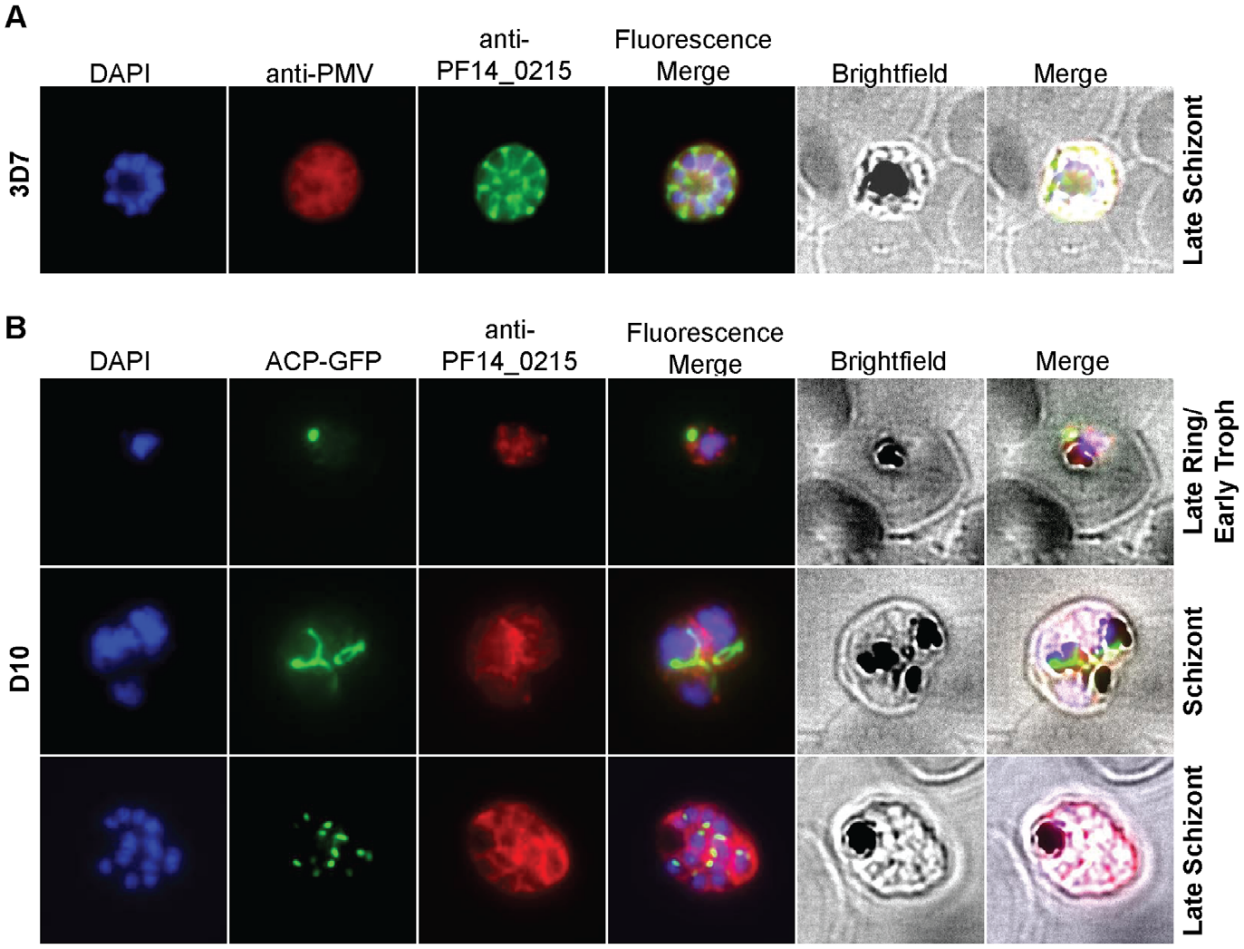
Cellular localization of PfHRD1. (**A**) *Pf*HRD1 and plasmepsin V (PMV), an ER membrane marker, co-localize in *P. falciparum*. (**B**) At various stages of the parasite life cycle, *Pf*HRD1 was co-stained with the nuclei (DAPI) and the apicoplast (ACP-GFP) in the *P. falciparum* strain D10.

We further investigated the possible role of PF14_0215 E3 ligase and determined its localization by immunofluorescence microscopy using custom antibodies. We first verified the specificity of the custom antibody against PF14_0215 (see figure S2). Then we used the custom antibody to immuno-stain PF14_0215, which was found to co-localize with Plasmepsin V, a *Plasmodium* protein known to reside in the ER membrane [26] (Figure 4A). Co-imaged with DAPI-stained nuclei and ACP-GFP, an apicoplast marker, for comparative purposes, PF14_0215 was observed at various stages of the parasite life cycle and was found within reticular structures outside the nuclear regions at the trophozoite and schizont stages of the parasite, similar to the physical attributes of the ER (Figure 4B). In the late schizont stage PF14_0215 proteins reside within globular structures surrounding each budding merozoite in a pattern typical of the ER (Figure 4B). These observations support that PF14_0215 E3 ubiquitin ligase resides in the ER membrane, consistent with a putative role in the ERAD pathway. To further confirm our findings, we analyzed PF14_0215 function by reverse genetics using a double recombination gene knock out approach (Figure S3). Despite multiple attempts, we were only able to obtain crossover events at the 3′ UTR of PF14_0215 and failed to produce viable parasites with a disrupted PF14_0215 gene suggesting that PF14_0215 is essential for parasite survival. This result evokes the observed essentiality of HRD1 in other eukaryotic model organisms. Evidences showing *in vitro* ubiquitylation activity, ER membrane localization, and gene essentiality strongly suggest that PF14_0215 is the E3 ligase HRD1 *Plasmodium* homologue of the classical ERAD system.

### PFL1245w and PFL0190w are Functional Cytosolic Ubiquitin Activating and Conjugating Enzymes

**Figure 5:**
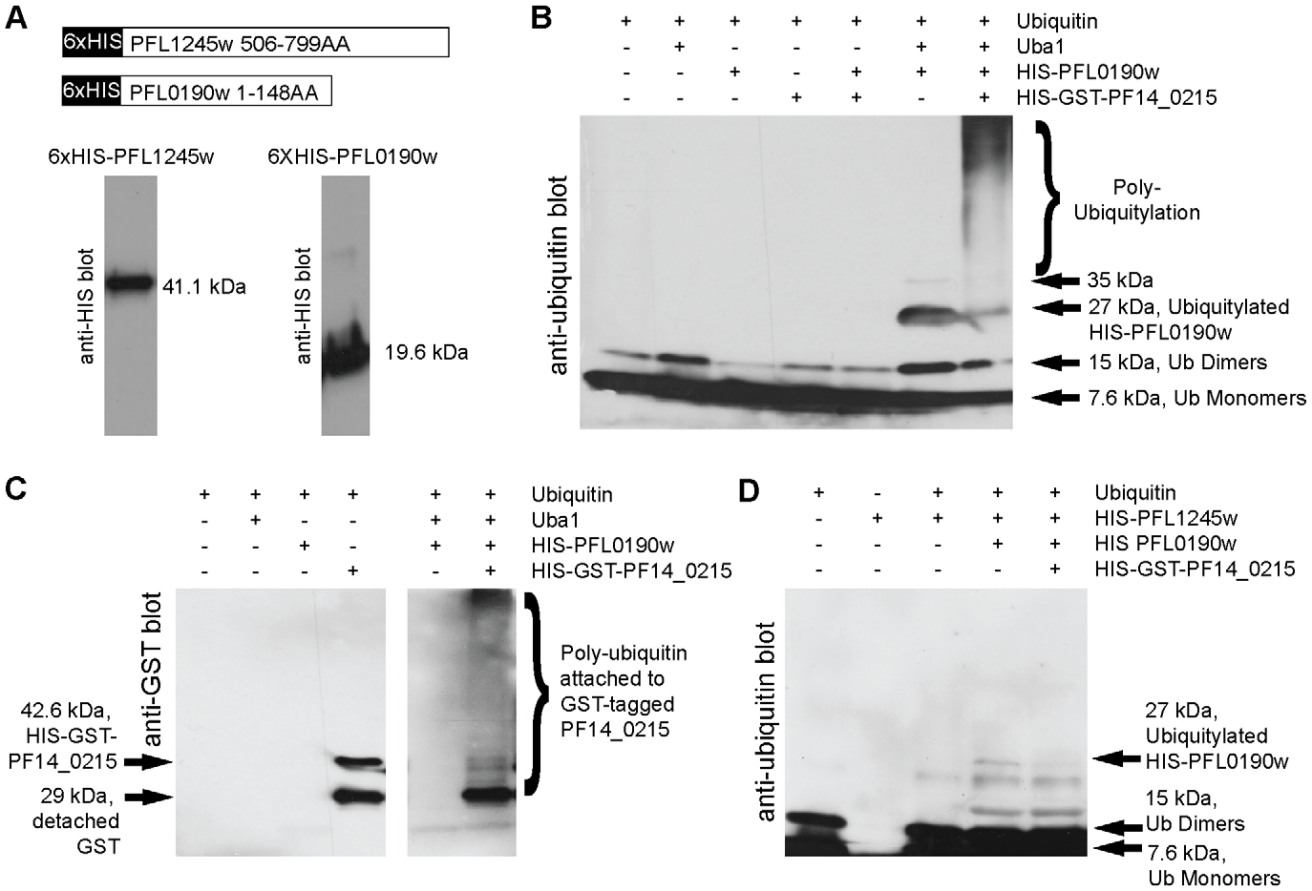
*In vitro* ubiquitylation assay of *Pf*UBA1 and *Pf*UBC. (**A**) 6xHIS-tagged recombinant *Pf*UBA1 (E1) and *Pf*UBC (E2) are depicted and anti-HIS blots reveal purification. (**B**) *In vitro* ubiquitylation assays were performed with recombinant *Pf*UBC (E2), which revealed an attachment of a single ubiquitin when incubated with human UBA1 (E1) (second lane from the right) and an increased number of ubiquitylation products when both UBA1 and recombinant *Pf*HRD1(E3) was added (far right lane). (**C**) When incubated with human UBA1 (E1), recombinant *Pf*UBC (E2) (along with the other necessary reagents) attaches polymers of ubiquitin to recombinant *Pf*HRD1 (E3) (far right lane). (**D**) Recombinant *Pf*UBA1 (E1) is capable of attaching a single ubiquitin to recombinant *Pf*UBC (E2), which is depicted by the appearance of a 27 kDa band (second lane from the right). Extra banding was not detected with the addition of recombinant *Pf*HRD1 (E3) (far right lane).

PFL1245w and PFL0190w are predicted to be the E1 ubiquitin-activating enzyme and the E2 ubiquitin-conjugating enzyme, respectively. In order to validate the *in silico* predictions, we have recombinantly cloned, expressed and purified PFL1245w and PFL1090w (Figure 5A and Figure S1) to test their *in vitro* activities. Since we found that PF14_0215 is likely the *Plasmodium* homologue of HRD1 of the ERAD system, we also tested the interaction of PFL1245w and PFL1090w with PF14_0215. Our results show that recombinant PFL0190w (19.6 kDa) is capable of *in vitro* mono (27 kDa band) and di-auto-ubiquitylation (35 kDa band) activity when incubated with commercially available human UBA1 (Figure 5B, second lane from the right). When the recombinant PF14_0215 RING domain was added to the reaction, a large laddering effect (poly-ubiquitin chains) was observed (Figure 5B, far right lane) similar to the effect observed using the commercial human UBCH5a (Figure 3B, third lane from the left). Anti-GST blots further revealed that these poly-ubiquitin chains were attached to the recombinant GST-tagged RING domain of PF14_0215 (Figure 5D). In addition, we immunoprecipitated the endogenous PF14_0215 E3 protein using our custom antibodies and were able to detect elevated amounts of ubiquitylated products when incubated *in vitro* with ubiquitylating reactions, which included our recombinant full-length PFL0190w E2 protein (Figure S4). These results show that PFL0190w is a functional E2 ubiquitin-conjugating enzyme that is compatible *in vitro* with endogenous PF14_0215.

*In vitro* ubiquitylation assays were repeated using recombinant parasite E1 PFL1245w together with E2 PFL0190w and E3 PF14_0215 (Figure 5D). PFL1245w was able to ubiquitylate PFL0190w as shown by the presence of a band at 27 kDa. When recombinant *Plasmodium* E3 ligase PF14_0215 was added to the reaction, no increase of ubiquitylating activity could be detected. Considering the mode of action of the classical ERAD-system, it is possible that the full-length of recombinant PF14_0215 as well as additional accessory proteins are required for a full *in vitro* activity of all three *Plasmodium* proteins. On the whole, these results validate *(i)* PFL1245w as a functional E1 ubiquitin-activating enzyme that can work *in vitro* with the E2 PFL0190w, and *(ii)* PFL0190w as a functional E2 ubiquitin-conjugating enzyme that can work *in vitro* with the E3 ligase PF14_0215.

**Figure 6:**
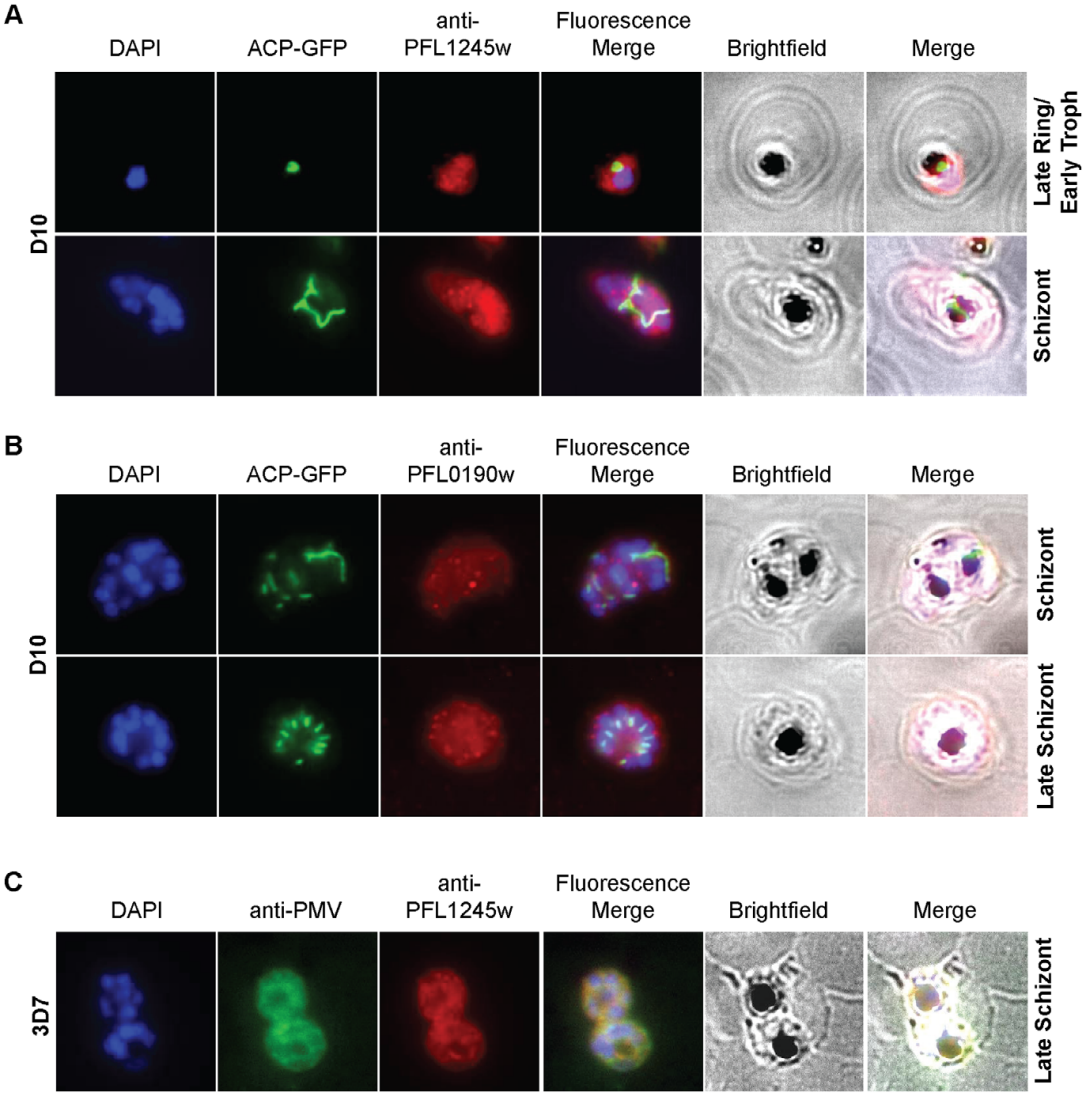
Localization studies of PfUba and Pfubc7. (**A,B**) IFA experiments, using different parasite stages, show that *Pf*UBA1 and *Pf*UBC localize mainly to the cytosol. (**C**) When co-immunostained with plasmepsin V (PMV), a *Plasmodium* ER membrane protein marker, there was noticeable overlap between the two proteins, suggesting *Pf*UBA1 recruitment to the ER as well.

To further validate PFL1245w and PFL0190w as the likely *Plasmodium* ERAD E1 and E2 enzymes, respectively, we investigated their localization using custom-made antibodies; antibody-specificity was verified by immunoblotting (see Figure S2). In other model organisms, the classical ERAD E1 UBA1 and E2 UBC7 proteins are reported to reside in the cytoplasm until they are needed and are recruited to the outer membrane of the ER. Using immunofluorescence microscopy, we found that both PFL0190w and PFL1245w are mainly dispersed throughout the cytoplasm with small aggregations scattered throughout the parasite (Figures 6A,B). In addition, when PFL1245w was co-immunostained with PMV, an ER membrane marker, there was noticeable overlap between the two proteins, which suggests that PFL1245w is likely recruited to the ER membrane as expected (Figure 6C).

The localization pattern of PFL1245w and PFL0190w indicates cytoplasmic residence with possible transient recruitment to the ER that is consistent the classical ERAD model. Given their *in vitro* activities in auto-ubiquitylating assays and their pattern of localization, PFL1245w and PFL0190w are likely the *Plasmodium* homologues for E1 UBA1 and E2 UBC7 involved in the parasite’s ERAD system.

## Discussion

Proteasome inhibitor studies have shown promising antimalarial results and have ignited interest in studying how protein degradation in *Plasmodium* can be targeted for effective drug discovery and synthesis. Though there have been several reports on the efficacy of various proteasome inhibitors on malaria parasites, there have been relatively few studies done on functionally characterizing the malarial protein degradation system upstream of the proteasome. An integral part of protein degradation within eukaryotic cells is the ERAD system, which relies on ubiquitylation in order to shuttle misfolded proteins across the ER membrane and label them for degradation by the 26S proteasome. Here, we show that ES_I_, a known ERAD inhibitor, is toxic to the *Plasmodium* parasite within low µM ranges and causes increased levels of ubiquitylated products. These results provide the first indications that an ubiquitin-dependent ERAD system does exist within the parasite and also gives a proof of concept that the ERAD system could be a point of antimalarial drug targeting.

**Figure 7:**
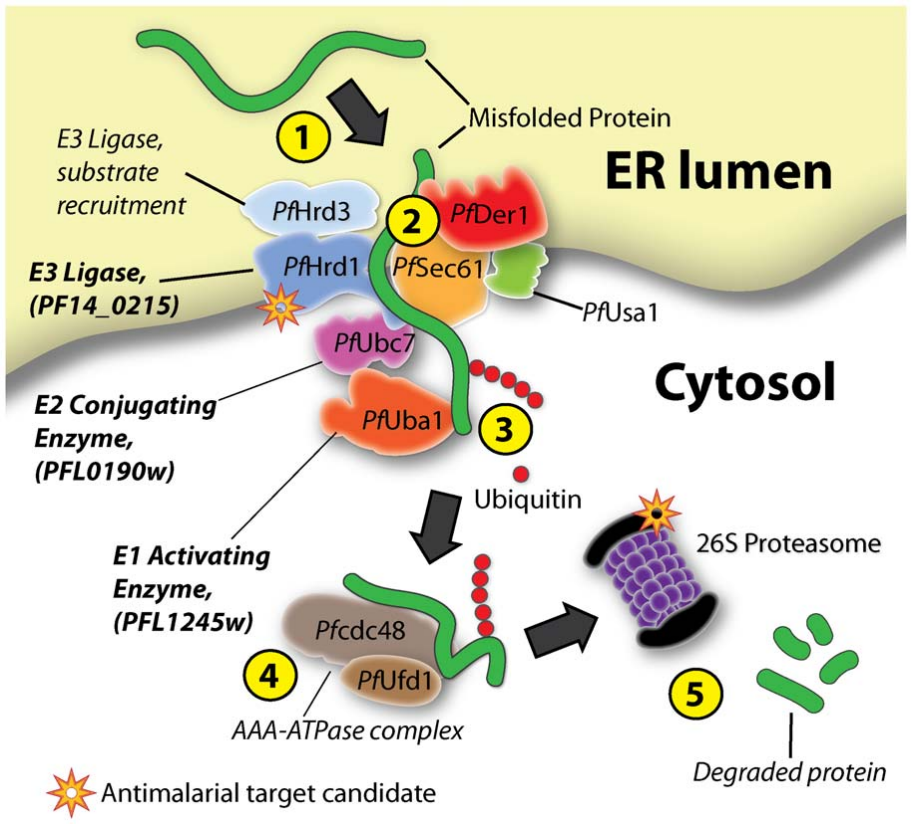
Graphical depiction of the *Plasmodium* ERAD pathway in protein quality control and its potential for antimalarial strategies. Similar to their human and yeast counterparts, the components of the *Plasmodium* ERAD system likely serve to recognize and translocate misfolded ER luminal proteins to the cytosol for degradation by the 26S proteasome. A simplified flowchart is presented: (1) misfolded proteins are recognized and recruited to the ERAD complex, (2) where they are inserted across the ER membrane through a pore formed by a complex of ERAD proteins. (3) During translocation, the aberrant proteins are poly-ubiquitylated by the concerted action of the E1 ubiquitin-activating enzyme *Pf*UBA1 (PFL1245w), the E2 ubiquitin-conjugating enzyme PfUBC (PFL0190w), and the E3 ligase *Pf*HRD1 (PF14_0215). (4) The ubiquitylated aberrant proteins are extracted by the CDC48-UFD1-NPL4 AAA-ATPase complex and shuttled to the 26S proteasome (5) for degradation. *Plasmodium* ERAD drug target candidates are highlighted with an orange star.

According to our bioinformatics analysis, *in vitro* ubiquitylation assays and localization studies, it seems that the *Plasmodium* ERAD system may function similarly to that of other eukaryotic model systems. The domain architecture of these putative ubiquitylating *Plasmodium* ERAD proteins are homologues to that of their identified counterparts in other eukaryotes [21], while localization studies show them localizing to their expected destinations of either the ER membrane or cytosol. The *in vitro* ubiquitylation assays performed on recombinant versions of PFL1245w, PFL09190w and PF14_0215 revealed that these proteins are capable of facilitating ubiquitylation and that they can work together. In many cases, E3 ligases with RING domains are known to mediate the transfer of ubiquitin between E2 conjugating enzymes and target substrates without actually binding to ubiquitin itself [27]. And though recent studies are reporting novel RING E3 ligase complexes (*e.g.* RBR ubiquitin ligases) are capable of binding to ubiquitin [28], autoubiquitylation of E3 ligases have been more associated with HECT E3 ligases rather than RING E3 ligases. However, when we experimentally studied the E3 RING domain of PF14_0215, we found that PF14_0215 was capable of autoubiquitylation, which is reminiscent to the yeast HRD1p E3 ligase, which has also been reported to autoubiquitylate as a mechanism to regulate itself [29], [30]. All together, our data indicates that PFL1245w, PFL0190w, and PF14_0215 are the *Plasmodium falciparum* UBA1, UBC and HRD1 (*Pf*UBA1, *Pf*UBC and *Pf*HRD1) respectively and serve to ubiquitylate misfolded ER proteins for translocation from the ER lumen to the cytosol for proteasome degradation (see figure 7 for a graphical model).

The results from the knock out attempts of PF14_0215 suggest that PF14_0215 is an essential *Plasmodium* gene. As part of the ERAD system, the probable essentiality of PF14_0215 is not surprising since the ERAD system, and protein degradation in general, is known to be an essential part of eukaryotic biology. That is why there is burgeoning interest in proteasome degradation and inhibitors in regards to antimalarial drug discovery. If the protein degradation at the proteasome level can be effectively exploited as a potential antimalarial target, it is reasonable to believe that upstream pathways, such as the ubiquitylating components within the ERAD system, may also serve as viable antimalarial drug targets. Currently, a wide range of ubiquitylating enzymes, from E1 activating enzymes to deubiquitylating enzymes (DUB)s are being screened for inhibitors that may confer anti-cancer properties. In fact, there are already several inhibitors of ubiquitin or ubiquitin-like enzymes that have shown effective results against cancer (Sun, 2003; Yang *et al*, 2007), with some already in clinical trials (for a review, see [31]).

Some may argue that targeting a highly conserved system such as the ERAD pathway in the *Plasmodium* is likely to have cross-reactivity with the very similar human host ERAD counterpart and is unlikely to produce a tight parasite-specific drug. However, inhibitors of ubiquitin E1 enzymes have already been demonstrated to reduce leukemia and multiple myeloma, while maintaining limited toxicity in mouse models [32]. The limited toxicity of E1 ubiquitin inhibitors observed in normal host cells is likely explained by the notion that inhibition of proteasome degradation has more of a profound effect on highly proliferative cells, such as malignant tumors and protozoan parasites, due to their higher metabolic needs. Furthermore, since it has been shown that E3 ligases are the most various and divergent within eukaryotes [21], higher parasite-specificity can be achieved by targeting essential parasite-specific E3 ligases. Currently, there are specific inhibitors of E3 ubiquitin ligases that are in clinical trials. For example, inhibitors of MDM2, the E3 ligase regulator of the tumor suppressor protein p53, has shown promising anti-myeloma activity [33]. In theory, components of the *Plasmodium* ERAD degradation pathway, particularly its E3 ligases (i.e. SEL1L/HRD3 [34], gp78 [35], RNF5/RMA1 [36], TEB4/March6 [37] and TRC8/RNF139 [38]), could produce an effective new strategy against *Plasmodium* as they are both specific and likely essential to the parasite life cycle. Interestingly, there have also been recent identifications of a duplicated ERAD-like system within the plastids of some apicomplexans, cryptomonads, and diatoms [24], [25], with *P. falciparum* included. However, instead of functioning in protein degradation, the plastid ERAD-like system is hypothesized to have roles in plastid protein import [39], which may also turn out to be an effective drug target.

The biology of the *Plasmodium* ERAD system and overall degradation pathways are worthy themes to investigate. It will be interesting to see whether or not these core ubiquitylating enzymes do collaborate in conjugating ubiquitin to misfolded proteins *in vivo* and work together with other known ERAD proteins (*i.e.* Der1, Cdc48, Ufd1) for targeted protein degradation. Also, whether or not protein degradation in protozoan parasites has unique features compared to human and yeast models. However, this can only be uncovered with more targeted and vigilant examination. One aspect of parasite biology that we can be certain of is that protein degradation is necessary for parasite survival and has already been shown to be a good candidate for antimalarial targeting with reports of proteasome inhibitors conferring *Plasmodium* IC_50_ values within the µM to nM ranges [8]. All together our results show that the *Plasmodium* ERAD system offers at least one parasite-specific drug target candidate, the ERAD E3 ubiquitin ligases, which have been characterized as being divergent from their human host counterparts. Though we have just begun to explore the machinery that is responsible for protein degradation in the human malaria parasite, we believe that the *Plasmodium* ERAD system may provide great drug target candidates that are both effective and parasite-specific.

## Materials and Methods

### Half Maximal Inhibitory Concentration (IC50) Assays and Ubiquitylated Protein Abundance Experiments with Eeryarestatin I

Eeryarestatin I (ES_I_) (EMD Millipore Corporation) was reconstituted at 10 mM in DMSO. 3D7 parasites were introduced to a 3-fold serial dilution of ES_I_ in complete culture medium in 96-well plates and incubated for 72 hours in a humid modular incubation chamber (Billups-rothenberg) after gassing at 1% O_2_/3% CO_2_/96% N_2_. Plates were frozen overnight then thawed and evaluated for relative parasite growth with SYBR Green dye and a Molecular Devices Gemini EM spectrofluorometer.

3D7 parasites were synchronized with sorbitol treatment. During the early trophozoite stages, parasites were drugged with either of four different conditions: ES_I_ (10 µM), MG132 (0.1 µM), ES_I_ (10 µM) + MG132 (0.1 µM) or DMSO only (equivalent volumes to the drug conditions). After 8 hours incubation, parasites were isolated with 0.15% saponin and crude protein lysates were extracted. 0.5 µg of protein lysates from each condition were loaded in each well for SDS-PAGE. Immunoblots were probed with rabbit anti-ubiquitin antibodies (Millipore; 1∶2500) and goat anti-rabbit antibodies conjugated to HRP (1∶5000; Pierce).

### *In silico* Discovery of Candidate Ubiquitylating Proteins and Protein Alignments

The translated genome of *P. falciparum* v8.0 was obtained from PlasmoDB (www.plasmodb.org) and was bioinformatically searched to identify ubiquitylating proteins using a previously published approach [21]. Domain architecture analysis was performed using InterProScanSequence [40]. Transmembrane domains were predicted with TMHMM [41]. Signal peptides were predicted with signal 3.0 [42]. Pictures were generated with Matlab®. Using MUSCLE [43], we performed protein alignments on PF14_0215, comparing it to the HRD1 of H. sapien (BAC24801.1), S. cerevisiae (NP_014630.1), S. pombe (NP_596376.1) and M. oryzae (XP_003709820.1).

### Cloning and Purification of Recombinant Proteins

The plasmid pGS-21a (GenScript) and a modified version of it (designed as pGS-21aHis) were used as cloning vectors. The region coding for a GST was removed so that only the two 6xHIS tags subsist. The GST tag, along with the tandem 6xHIS tag, was removed by cutting pGS-21a with ClaI and NcoI. A new 6xHis-coding fragment was then amplified by PCR from purified pGS-21a using the primers GATCGAGATCGATCTCGATC and ATCCATGGCCTTACCGCTGCTATGATGATGAT and inserted to replace the GST using ClaI and NcoI. The sequence coding for the RING domain in PF14_0215 was PCR-amplified from 3D7 strain cDNA using the primers AGGGGATCCCTTAAGCCGCGGCCTTTACATATGACAGCAGAT and AGGAAGCTTTTAACTAGTGCTAGCTTACTTTTGTGTTGTATCATTTTCTG. A sequence segment that contains the E1 ubiquitin-activating domain of PFL1245w was PCR-amplified using the primers AGGGGATCCCTTAAGCCGCGGGTGGTGAATATTTTTGGGTTGG and AGGAAGCTTTTAACTAGTGCTAGCCCAACCCAAAAATATTCACCAC. The entire gene of PFL0190w was amplified from 3D7 strain cDNA with the primers AGGGGATCCCTTAAGCCGCGGATGGCCCTTAAAAGAATAACAAAA and AGGAAGCTTTTAACTAGTGCTAGCTTATTGTGCATATTTTTGTGTCC. The amplified fragments were digested with SacII and SpeI and ligated into pGS-21a (6xHis-GST) or pGS-21aHis (6xHis only) where the gene was N-terminally tagged. Constructs were inserted into *E. coli* for expression.

Transformed *E. coli* cells were grown in LB at 25°C until OD_600_>0.5. IPTG 1 mM was added to the cultures, which were then incubated overnight at 12°C. Cells were collected by centrifugation at 14,000 g and 4°C and resuspended in lysis buffer (Tris-HCl pH 7.5 25 mM, NaCl 500 mM, Triton X-100 1% (v/v), Mini EDTA-free Protease Inhibitor Cocktail (Roche), and AEBSF 1 mM). Cells were sonicated and lysates were cleared by centrifugation at 14,000 g and 4°C. Purifications of GST-tagged proteins were performed on glutathione agarose beads (Sigma-Aldrich). Glutathione-bound proteins were washed three times with GST wash buffer (Tris-HCl pH 7.5 25 mM, NaCl 300 mM, Triton X-100 1% v/v) and eluted with GST elution buffer (Tris-HCl pH 7.5 25 mM, NaCl 150 mM, reduced glutathione 15 mM, Triton X-100 0.01% v/v, glycerol 40% v/v). Purifications of His-tagged proteins were performed on Ni-NTA beads (Qiagen). Beads-bound proteins were washed several times with wash buffer (Tris-HCl pH 7.5 25 mM, NaCl 500 mM, imidazole 30 mM, glycerol 5% v/v) and subsequently eluted with elution buffer (Tris-HCl pH 7.5 25 mM, NaCl 500 mM, imidazole 250 mM, glycerol 5%). Purified protein extracts were analyzed by immunoblots. GST-tagged proteins were detected using a goat anti-GST antibody (1∶5000; GE Healthcare) and a donkey anti-goat antibody conjugated to horseradish peroxidase (1∶20,000; Jackson Immuno research). His-tagged proteins were detected using a mouse anti-His antibody (1∶2500; Millipore) and a goat anti-mouse antibody conjugated to horseradish peroxidase (1∶10,000; BioRad).

### Custom Primary Antibodies

The following peptide sequences were used as antigens for the production of affinity-purified antibodies (Fisher Scientific or Genscript) in rabbits: RFKSFQKYRELTKNIETK for PF14_0215, KTDRTKYHQTAKAWTQKYAQ for PFL0190w, CSDQDLVDVLIPSIQFIYK for PFL1245w.

### Parasite Culture and Transfection

The *P. falciparum* 3D7 (MRA-102, MR4) and D10 ACP(transit)-GFP (MRA-568, MR4) strains were grown in human O^+^ red blood cells according to a previously described protocol [44]. Transfections were carried out by electroporation of parasite-infected red blood cells as described in [45]. Successful transfectants were obtained by positive selection with WR99210 [46], and, when relevant, negative selection with 5-fluorocytosine [47].

### Immunofluorescence Assay

Immunofluorescence assays were performed using the protocol described in [25]. Briefly, cells were washed with PBS and fixed for 1 h at 37°C in PBS containing 4% of paraformaldehyde and 0.0075% glutaraldehyde. Fixed cells were then pelleted by centrifugation at 700 g for 2 min and washed once for 10 minutes with glycine 1.25 M in PBS. After another centrifugation at 700 g for 2 min, the pelleted cells were permeabilized for 10 min with TritonX-100 0.1% (v/v) in PBS. Permeabilized cells were then washed once for 10 minutes with glycine 1.25 M in PBS and pelleted by centrifugation (700 g for x min). After a minimum of a 1 h-long incubation in BSA 3% (w/v) in PBS, the desired target-specific custom primary antibody was added for at least 2 hours at room temperature or overnight at 4°C at a dilution of 1∶200. Cells were subsequently extensively washed with PBS. Secondary goat anti-rabbit IgG Alexa Fluor® 488 or donkey anti-rabbit IgG Alexa Fluor® 568 were then added according to their respective primary antibodies at a dilution of 1∶100 for two hours at room temperature. Cells were washed 3 times with PBS and DAPI stain was finally added at 100 ng/mL final concentration for *nuclei* visualization. Anti-plasmepsin V antibodies (MRA-815A, MR4) were used to visualize the endoplasmic reticulum membrane [26].

Microscopy slides were mounted with slow-fade mounting medium (Fluoromount-G; Southern Biotech) and viewed with an Olympus BX40 fluorescence microscope using a 100X objective lens (UPlanFI). Image capture was done with a CoolSNAP cf camera and processed by the Metavue software (Photometrics). Images were finally merged and background was reduced using ImageJ software.

### *In vitro* Ubiquitylation Assay

A mix containing free ubiquitin 50–200 µM (Boston Biochem), Human recombinant UBE1 E1 enzyme 0.05-0.2 µM (Boston Biochem), Human recombinant UBC E2 enzyme 1–5 µM (Boston Biochem), and purified *Plasmodium* E3 ligase 1–12.5 µM was incubated in reaction buffer (Tris-HCl pH 7.4 50 mM, DTT 1 mM, re-energizing buffer from Boston Biochem) for 2 h at 37°C. Alternatively, Human E1 and/or E2 were replaced with our purified *Plasmodium* proteins. Reactions were then analyzed by SDS-PAGE and anti-ubiquitin or anti-GST immunoblotting. Anti-ubiquitin immunoblots were probed with rabbit anti-ubiquitin antibodies (1∶2500; Millipore) and goat anti-rabbit antibodies conjugated to HRP (1∶5000; Pierce). Anti-GST immunoblots were probed with goat anti-GST antibodies (1∶2500; GE Healthcare) and donkey anti-goat antibodies conjugated to HRP (1∶5000; Jackson Immunoresearch).

### Gene Disruption Experiments

Double recombination-based gene disruption plasmids were constructed with the 5′ UTR and 3′UTR of PF14_0215 flanking a human dihydrofolate reductase (hDHFR) resistance cassette (for positive selection with WR99210) within the pCC1 vector that also contains a *Saccharomyces cerevisiae* cytosine deaminase (ScCD) cassette for subsequent negative selection [42]. Gene disruption vectors were transfected into Pf3D7. Successful integration was verified by PCR.

### Immunoprecipitation and *in vitro* Ubiquitylation Assay of Pulled-down Proteins

Isolated 3D7 parasites were resuspended in 20 mM HEPES pH7.9, 10 mM KCl, 1 mM EDTA, 1 mM EGTA, 1 mM DTT, 0.5 mM AEBSF (Fisher Scientific), 0.65% Igepal v/v and cocktail protease inhibitor (Roche) (lysis buffer 1). Parasites were allowed to lyse on ice for 10 minutes and then were spun down. Supernatant was collected. The pellet was then subsequently resuspended in 20 mM HEPES pH 7.9, 0.1 M NaCl, 0.1 mM EDTA, 0.1 mM EGTA, 1.5 mM MgCl_2_, 1 mM DTT, 1 mM AEBSF and cocktail protease inhibitor (Roche) (lysis buffer 2), and then incubated at 4°C with vigorous shaking for 20 minutes. After spinning, the supernatant was collected. The remaining pellet was then resuspended in lysis buffer 1 and then sonicated and spun down. Supernatant was collected and pooled with the previous two collected supernatants to create a mixture of extracted proteins. Extracted proteins were precleared with magnetic Protein A beads (Millipore) and then incubated with anti-*Pf*HRD1 (PF14_0215) antibodies for 2 hours at 4°C with gentle shaking. Pre-washed magnetic Protein A beads were added to the samples and allowed to incubate overnight at 4°C with gentle shaking. Samples were washed several times with lysis buffer 1. Pulled-down PF14_0215 proteins were left bound to the beads.

Human recombinant UBE1 (Boston Biochem), *Plasmodium* recombinant *Pf*UBC (PFL0190w) and pulled-down *Pf*HRD1 were added to a solution containing 0.2 M HEPES pH7.9, 50 mM DTT, re-energizing buffer (Boston Biochem), 0.5 µg/µL final concentration of biotin conjugated ubiquitin (Boston Biochem), 0.5 µg/µL final concentration of ubiquitin (Boston Biochem), 5 mM AEBSF (Fisher Scientific) and cocktail protease inhibitor (Roche). The reactions were allowed to incubate at 37°C with gentle agitation for two hours. After incubation, reactions were terminated with Laemmli buffer and incubated at 95°C for 5 mins. Activity was visualized with affinity blots. Biotin affinity blots were probed with streptavidin conjugated with peroxidase (1∶10,000; Jackson Immunoresearch). Immunoblot intensities were measured with ImageJ64 software.

## Supporting Information

Figure S1 **Coomassie-stained SDS-PAGE gels of purified recombinant proteins.** Coomassie-stained SDS-PAGE gels show the step-by step purification of recombinant (**A**) PF14_0215, (**B**) PFL1245w, and (**C**) PFL0190w that have been either tagged with HIS or with GST and HIS both. In each elution, a distinguishable band that represents the respective purified recombinant protein can be found at their expected molecular sizes: GST-HIS-PF14_0215(249–358 AA), 42.6 kDa; HIS-PFL1245w (506–799 AA), 41.4 kDa; HIS-PFL0190w (1–148 AA), 19.6 kDa.
(TIF)

Figure S2 **Immunoblots showing custom antibody specificity.** (**A**) The specificity of the custom-made antibody raised against PF14_0215 protein (59.8 kDa) was verified by western blot on crude parasite extract. (**B**) Using anti-PFL1245w custom antibodies on crude parasite protein extracts, a band at 131.8 kDa was detected on an immunoblot, which is the expected size for the PFL1245w protein. (**C**) Immunoblots were made using anti-PFL1245w custom antibodies on crude parasite protein extracts. A band at 131.8 kDa was detected, which is the expected size for the PFL1245w protein.
(TIF)

Figure S3 **Knock-Out Attempt for PF14_0215.** A PF14_0215 knockout vector was constructed with a human dihydrofolate reductase (hDHFR) selection cassette that is flanked by the 5′ UTR and 3′ UTR of the PF14_0215. A *Saccharomyces cerevisiae* cytosine deaminase (ScCD) cassette (not shown) was placed outside the PF14_0215 5′ UTR and 3′ UTR sections to be used for negative selection. Primers (indicated by red arrows) were designed to only amplify crossover events at either the 5′ or 3′ UTR of PF14_0215. PCR experiments show that only parasites that underwent a 3′ UTR crossover event, that still leaves the PF14_0215 intact, could be recovered. Double recombination that would excise the endogenous PF14_0215 gene was never recovered.
(TIF)

Figure S4 ***In vitro*** **ubiquitylation activity of immunoprecipitated** ***Pf*** **HRD1.** Using anti-*Pf*HRD1 (PF14_0215) antibodies, endogenous *Pf*HRD1 was immunoprecipitated from 3D7 strains. *In vitro* ubiquitylation assays were performed using biotinylated-ubiquitin, thus only newly ubiquitylated products can be detected with streptavidin blots. Human recombinant UBE1 (lane 1) by itself was able to produce a single ubiquitylated product when incubated with biotin-ubiquitin. Aggregates of biotinylated ubiquitin (depicted by the arrow) were seen in all lanes (even without the addition of any ubiquitylating enzymes, not shown) and are considered as background. Recombinant *Pf*UBC (PFL0190w) (lane 2) and immunoprecitated *Pf*HRD1 (lane 3) by themselves were unable to produce any ubiquitylated products. UBE1 and *Plasmodium Pf*UBC together (lane 4) were able to generate significant levels of newly ubiquitylated products. With the addition of immunoprecipitated *Pf*HRD1 (lane 5), we were able to detect around a 44% increased level of ubiquitylation when compared to lane 4.
(TIF)
