# Supplementary figures and images for: Characterization of the Ubiquitylating Components of the Human Malaria Parasite’s Protein Degradation Pathway
Source: PLoS One. 2012 Aug 17;7(8):e43477. doi: 10.1371/journal.pone.0043477 (PMC3422240; doi:10.1371/journal.pone.0043477)

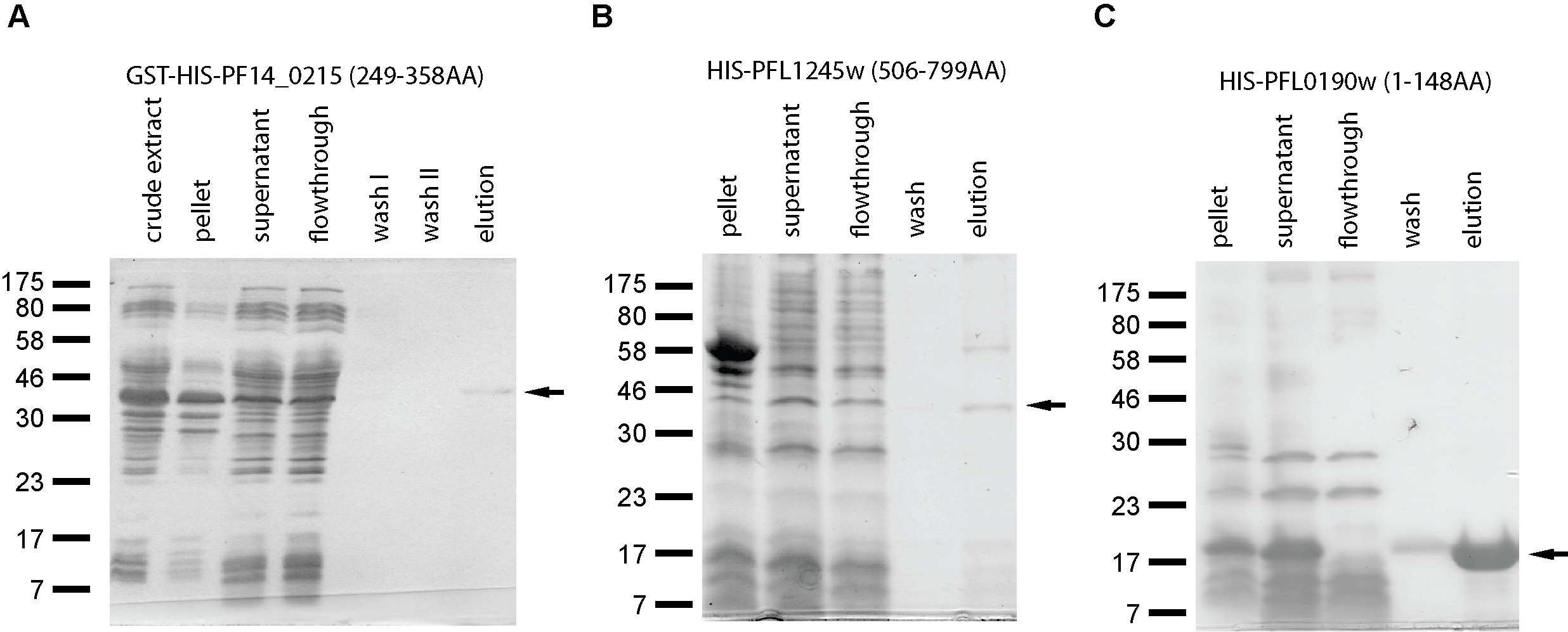

Supplement: Figure S1 — Coomassie-stained SDS-PAGE gels of purified recombinant proteins. Coomassie-stained SDS-PAGE gels show the step-by step purification of recombinant (A) PF14_0215, (B) PFL1245w, and (C) PFL0190w that have been either tagged with HIS or with GST and HIS both. In each elution, a distinguishable band that represents the respective purified recombinant protein can be found at their expected molecular sizes: GST-HIS-PF14_0215(249–358 AA), 42.6 kDa; HIS-PFL1245w (506–799 AA), 41.4 kDa; HIS-PFL0190w (1–148 AA), 19.6 kDa. (TIF) [file pone.0043477.s001.tif]

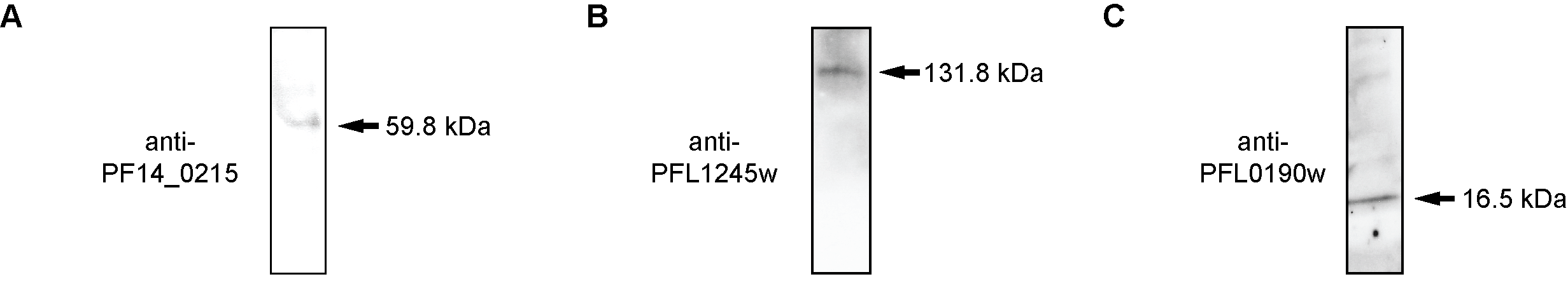

Supplement: Figure S2 — Immunoblots showing custom antibody specificity. (A) The specificity of the custom-made antibody raised against PF14_0215 protein (59.8 kDa) was verified by western blot on crude parasite extract. (B) Using anti-PFL1245w custom antibodies on crude parasite protein extracts, a band at 131.8 kDa was detected on an immunoblot, which is the expected size for the PFL1245w protein. (C) Immunoblots were made using anti-PFL1245w custom antibodies on crude parasite protein extracts. A band at 131.8 kDa was detected, which is the expected size for the PFL1245w protein. (TIF) [file pone.0043477.s002.tif]

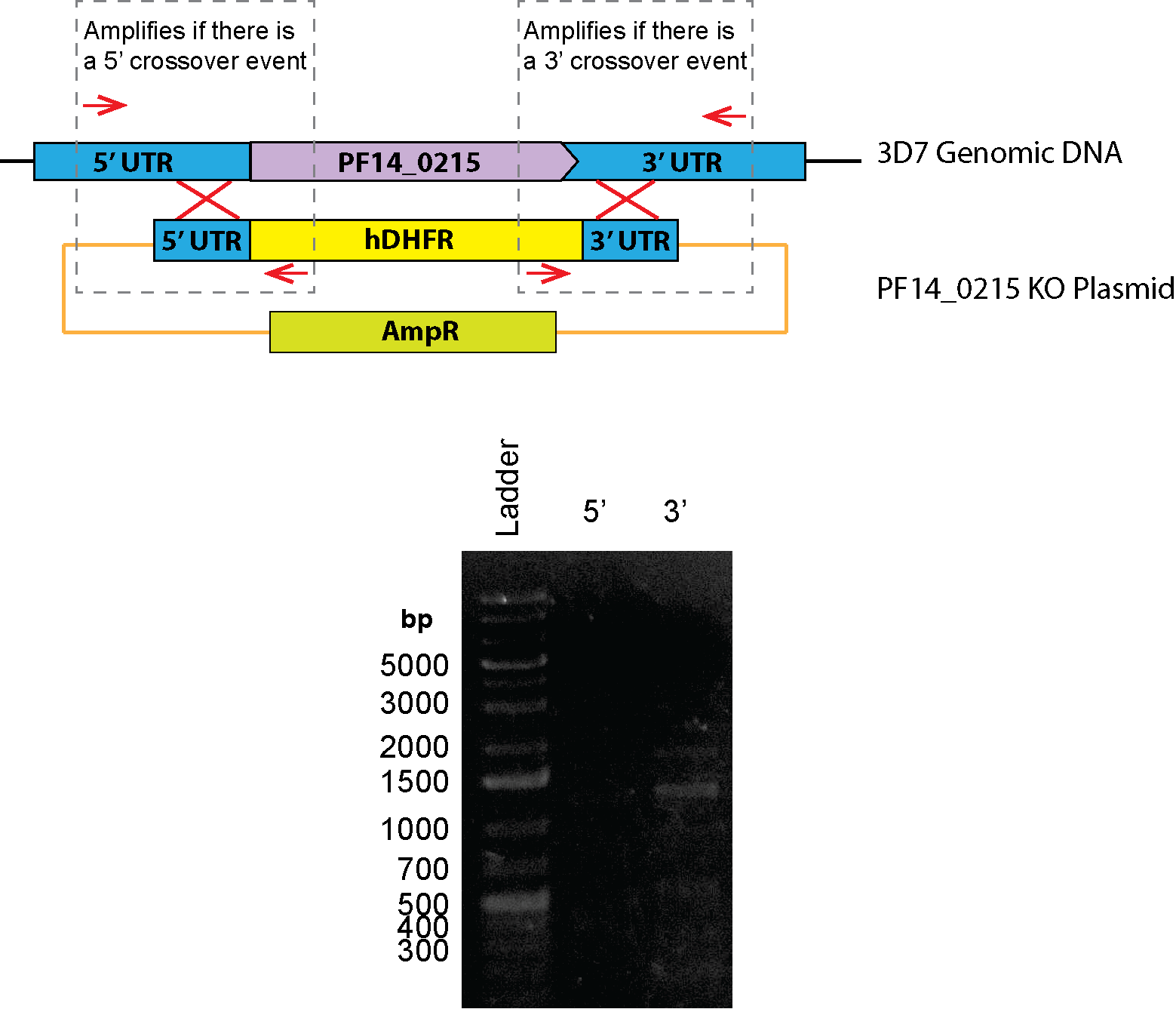

Supplement: Figure S3 — Knock-Out Attempt for PF14_0215. A PF14_0215 knockout vector was constructed with a human dihydrofolate reductase (hDHFR) selection cassette that is flanked by the 5′ UTR and 3′ UTR of the PF14_0215. A Saccharomyces cerevisiae cytosine deaminase (ScCD) cassette (not shown) was placed outside the PF14_0215 5′ UTR and 3′ UTR sections to be used for negative selection. Primers (indicated by red arrows) were designed to only amplify crossover events at either the 5′ or 3′ UTR of PF14_0215. PCR experiments show that only parasites that underwent a 3′ UTR crossover event, that still leaves the PF14_0215 intact, could be recovered. Double recombination that would excise the endogenous PF14_0215 gene was never recovered. (TIF) [file pone.0043477.s003.tif]

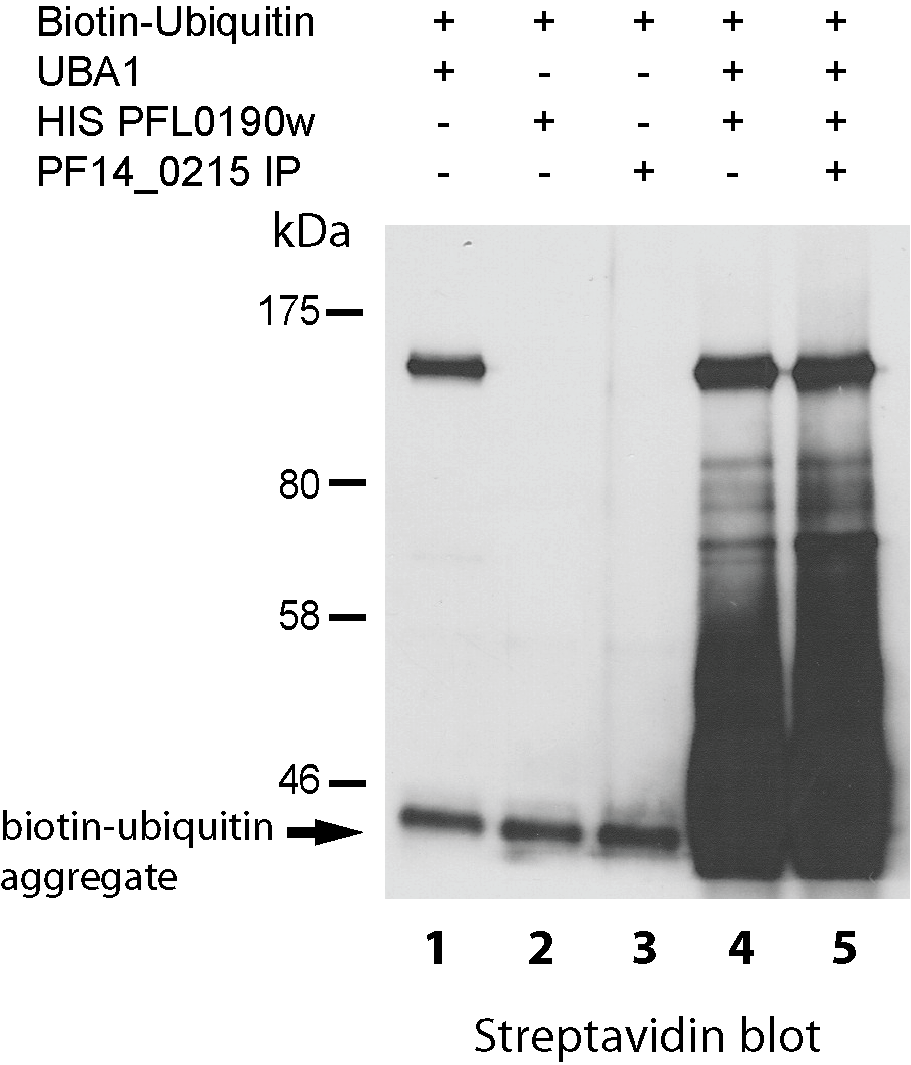

Supplement: Figure S4 — In vitro ubiquitylation activity of immunoprecipitated Pf HRD1. Using anti-PfHRD1 (PF14_0215) antibodies, endogenous PfHRD1 was immunoprecipitated from 3D7 strains. In vitro ubiquitylation assays were performed using biotinylated-ubiquitin, thus only newly ubiquitylated products can be detected with streptavidin blots. Human recombinant UBE1 (lane 1) by itself was able to produce a single ubiquitylated product when incubated with biotin-ubiquitin. Aggregates of biotinylated ubiquitin (depicted by the arrow) were seen in all lanes (even without the addition of any ubiquitylating enzymes, not shown) and are considered as background. Recombinant PfUBC (PFL0190w) (lane 2) and immunoprecitated PfHRD1 (lane 3) by themselves were unable to produce any ubiquitylated products. UBE1 and Plasmodium PfUBC together (lane 4) were able to generate significant levels of newly ubiquitylated products. With the addition of immunoprecipitated PfHRD1 (lane 5), we were able to detect around a 44% increased level of ubiquitylation when compared to lane 4. (TIF) [file pone.0043477.s004.tif]
